# The BioAssay network and its implications to future therapeutic discovery

**DOI:** 10.1186/1471-2105-12-S5-S1

**Published:** 2011-07-27

**Authors:** Jintao Zhang, Gerald H Lushington, Jun Huan

**Affiliations:** 1 Center for Bioinformatics, University of Kansas, Lawrence, KS 66045, USA; 2 Molecular Graphics & Modeling Lab, University of Kansas, Lawrence, KS 66045, USA; 3 Department of Electrical Engineering & Computer Science, University of Kansas, Lawrence, KS 66045, USA

## Abstract

**Background:**

Despite intense investment growth and technology development, there is an observed bottleneck in drug discovery and development over the past decade. NIH started the Molecular Libraries Initiative (MLI) in 2003 to enlarge the pool for potential drug targets, especially from the “undruggable” part of human genome, and potential drug candidates from much broader types of drug-like small molecules. All results are being made publicly available in a web portal called PubChem.

**Results:**

In this paper we construct a network from bioassay data in PubChem, apply network biology concepts to characterize this bioassay network, integrate information from multiple biological databases (e.g. DrugBank, OMIM, and UniHI), and systematically analyze the potential of bioassay targets being new drug targets in the context of complex biological networks. We propose a model to quantitatively prioritize this druggability of bioassay targets, and literature evidence was found to confirm our prioritization of bioassay targets at a roughly 70% accuracy.

**Conclusions:**

Our analysis provide some measures of the value of the MLI data as a resource for both basic chemical biology research and future therapeutic discovery.

## Background

Intense growth in drug development investment in the past decade has not yet produced significant progress on the discovery of novel drugs and the validation of new drug targets: the average number of novel drugs entering the global market each year has remained roughly constant (approximately 26), along with only about 6-7 new drug targets introduced annually [1-3]. Moreover, the success rate of translating new drug candidates into US Food and Drug Administration (FDA)-approved drugs significantly decreased [4], mainly due to lack of efficacy and discovered adverse drug reactions, each of which accounts for 30% of late-stage drug failures in clinical development [5]. The increasing rate of drug attrition challenges the traditional drug design paradigm and makes the current “one gene, one drug, and one disease” assumption [6] questionable.

Under these circumstances, NIH launched the Molecular Libraries Initiative (MLI) in 2003 for identifying chemical probes to enhance the chemical biology understanding of therapeutically interesting genes and pathways [7], and for the purpose of expanding availability, flexibility, and utility of small-molecule bioassay screening data. The MLI especially focuses on genes in the “undruggable” part of human genome that has not been well investigated in private sectors for identifying their functions and potential therapeutics [7]. The MLI has also been synthesizing and screening much broader types of compounds for increasing the diversity of selecting potential drug candidates in chemical space (i.e., the set of all possible small organic molecules). All MLI results are being made freely available to researchers in both public and private sectors via a web portal called PubChem (http://pubchem.ncbi.nlm.nih.gov) [8]. PubChem provides valuable chemical genomics information in studying genes, pathways, cells and diseases, however, these data are noisy, high dimensional, with large volume, and contain outliers and errors. For instance, the activity score, which measure the biological activity level of screened compounds, are normalized in many different approaches without a consensus. Hence PubChem data deserve a careful investigation for the related research communities.

There are numerous criteria that chemical biologists might place on an initiative that aims to foster new paradigms in therapeutic discovery. Some obvious target-related benchmarks should include whether the assay priorities focus on the systems that stand a good chance of being potential druggable targets in their own right (i.e., share favorable attributes with known drug targets, and augment current biomedical capabilities). Our assessment of MLI bioassay targets thus focuses on a variety of their attributes relative to known drug targets. Given our interest in analyzing specific relationships with phenotypically interesting pathways, we choose to focus our target analysis strictly on target-based assays, whose target proteins are referred to herein as bioassay targets. As our basis for contrasting targets, we use target protein interaction profiles in the human PPI network, to evaluate whether the bioassay target selection was progressing effectively toward augmenting existing chemical genomics knowledge.

Biological network analysis approaches have gained popularity for organizing complex biological systems so that data retrieval, analysis, and visualization can be highly efficient. It can also reveal important biological patterns and functions that are deeply hidden in mass data repository. For instance, Stelzl *et al.*[*9*] used the Y2H system to generate and analyze a human PPI network, and calculated many interesting and critical patterns and characteristics. This was viewed as an important step toward the complete human protein-protein interactome. Chaurasia *et al.*[10,11] built a comprehensive web platform - the Unified Human Interactome database (UniHI, http://www.unihi.org), for querying and accessing human protein-protein interaction (PPI) data. The latest update of UniHI includes over 250,000 interactions between 22,300 unique proteins collected from 14 major PPI sources [11]. Using association data of approved drugs and drug targets obtained from the DrugBank database [12,13], Yildirim *et al.*[*2*] built a bipartite network composed FDA-approved drugs and their drug targets, which was an important step toward the complete characterization of the global relationship between protein targets of all drugs and all disease-gene products in the human protein interactome. Quantitative topological analyses of this drug-target network revealed that the targets of current drugs are highly overlapped and new drugs tend to bind previously targeted proteins [2]. In addition, based on disease genes data from the Online Mendelian Inheritance in Man (OMIM) database [14], Goh *et al.*[*15*] built a bipartite human disease network, and then generated two biologically relevant network projections: human disease network and disease gene network. This network-based approach revealed that genes associated similar disorders are more likely to have interactions between their products and higher expression profiling similarity between their transcripts, indicating the existence of disease-specific functional modules. In this study we use network-based analysis approaches to integrate PubChem data and existing research results on drug-target network [2], human diseasome [15], human protein interactome [9] for a better understanding of the correlations and interrelationships between disease genes, genetic disorders and drugs by (1) constructing a bioassay network for data in PubChem, visualizing complex data in a network view and characterizing the network using a variety of statistical tools, (2) mapping bioassay targets into the human PPI networks, and investigating the interrelationships between bioassay targets, and drug targets, disease genes, and essential genes, and (3) qualitatively analyzing the potential of bioassay targets to be potential therapeutic targets, quantitatively modeling this potential, and confirming our results using literature survey. Our analyses should provide some measures of the value of the MLI data as a resource for both basic chemical biology research and future therapeutic discovery.

## Results and discussion

We download all bioassay screening data from the PubChem BioAssay Database. As of January 2009, 1306 bioassays (1,126 with at least one active compound) and more than 30 million compounds have been deposited into PubChem by a variety of screening centers, and the size of PubChem data keeps increasing continuously. For each bioassay, tens to hundreds of thousand of compounds are tested either against specific target proteins in vitro or within a cell for investigating disease-related mechanisms. There are totally 151,930 compounds that are active in at least one bioassay, and 555,859 bioassay-active compound pairs across all the bioassays. On average each active compound is active in 3.7 bioassays, and each bioassay has 493.7 active compounds. Therefore a very sparse bipartite network of bioassays and compounds can be observed. In addition, 680 bioassays are found associated with at least one target protein and are considered target-based, and the rest are hence assumed cell-based bioassays. Moreover, we have found 289 distinct protein GI (gene identifier) numbers for all target-based bioassays, and 215 of them have official associated gene symbols.

### Generating and characterizing the BioAssay Network

**Figure 1:**
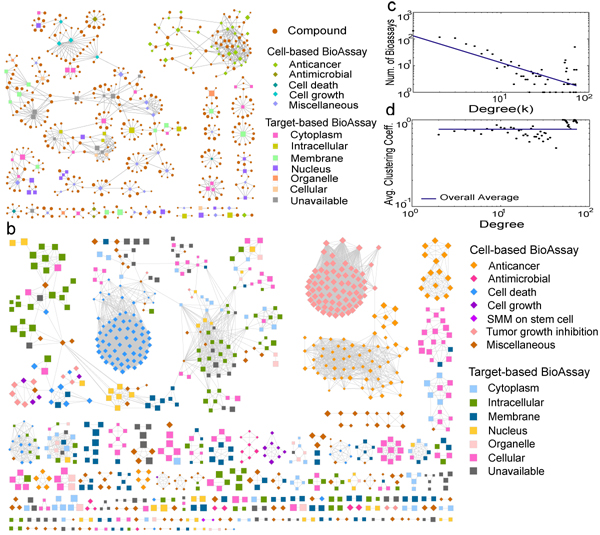
**The BioAssay network and topological distributions.** The bioassay networks and topological distributions. (a) A small subnetwork extracted from the complete bioassay-compound network. The size of each compound (bioassay) is proportional to the number of its active bioassays (compounds), cell-based bioassays are colored according to their screening purposes, and target-based bioassays are colored according to their cellular components. (b) The bioassay network in which nodes are bioassays and two bioassays are connected if they share at least 10% active compounds. The size of each node is proportional to the number of its active compounds, and the coloring of nodes is similar. (c) Distribution of the network node degrees. The power-law fitting clearly shows that is a typical scale-free network. (d) Distribution of the average clustering coefficients. The almost constant fitting shows the bioassay network is not hierarchical, not as other biological networks.

The complexity of PubChem data reveals that deep investigation will be difficult without organizing and visualizing the data in a rational matter, e.g., a bipartite network of bioassays and compounds. We first extract a small subnetwork from the complete bioassay-compound network by limiting the degrees of bioassay nodes and of compound nodes in the range of 20-40 and 10-20, respectively, resulting in a bipartite subnetwork with 127 bioassays, 457 compounds, and 842 links between them, as shown in Fig. 1a. There were a giant cluster and a few big clusters, and each cluster is tended to be composed of bioassays with the same purposes or cellular components. To visualize PubChem bioassay data with reasonable complexity, we generate a bioassay network projection from the bipartite bioassay-compound network as a complementary, bioassay-centered view, where bioassays are nodes and two bioassays are connected if they share similar binding profiles. We use *Jaccard coefficient* (the fraction of active compounds shared by two bioassays in the total number of distinct active compounds of them) to measure the similarity of bioassay binding profiles. By connecting any two bioassays that shared at least 10% active compounds, we generate a network of bioassays with 899 nodes and 6,080 edges as shown in Fig. 1b. Cell-based bioassays are represented by circles and colored according their screening purposes, while target-based bioassays are represented by rectangles and colored by their cellular components from the Gene Ontology database. From Fig. 1b, we find that there do exist a few clusters that contain both target-based and cell-based bioassays, while most clusters in the network have nodes of the same bioassay type (either target-based or cell-based). According to the definition of an edge in this network, such heterogeneous clusters reveal that the binding profiles of some target-based and some cell-based bioassays are to some extent similar, which can be helpful on understanding the protein-chemical interactions within the cell-based bioassays and possibly identifying critical proteins in the cell-based bioassays. Based on the bioassay network in Fig. 1b, we calculate the degree distribution *P*(*k*) of bioassays, measuring the probability of a given bioassay connects with other *k* bioassays (Fig. 1c). Excluding some outliers, the degree distribution decreases slowly as the degree increases and follows a power law with exponent = -1.009, and the fitting correlation coefficient as 0.955 and *R*^2^ = 0.527. This is a typical scale-free network according to the definition by Barabasi *et al.*[*16*], in which a small fraction of nodes have most of the linked connected, and the majority of nodes have only a few links, as observed in Fig. 1a. In addition, we also compute the distributions of the average clustering coefficient (Fig. 1d), and find that it is approximately independent on the node degree, and fluctuated around the mean 0.78 (standard deviation 0.14) as the degree increased. This answers that our bioassay network was scale-free, but not a hierarchical network [17], although typical biological networks were usually both scale-free and hierarchical. One reason could be that the edges in the bioassay network have no clear biological meaning.

### BioAssay targets in human protein-protein interaction network

**Figure 2:**
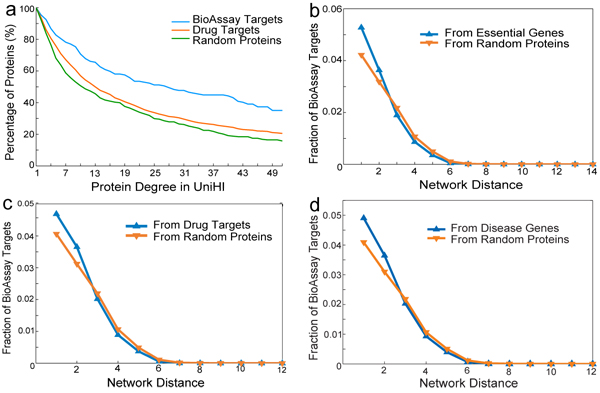
**Characteristics of BioAssay targets.** Characteristics of the bioassay network, including the degree distributions of bioassay targets, drug targets, and random proteins (a), followed by the distributions of the fractions of bioassay targets around (b) essential genes, (c)

The distributions of drug targets surrounding bioassay targets are of importance to examine the global relationships between them in the human PPI network, and to gain understanding of the potential of bioassay targets being promising new drug targets. We map bioassay targets and known drug targets into the UniHI network [10,11], identify 228 and 1,339 entries for them, respectively, and then calculate the median degree of each bioassay target and each drug target in UniHI. We also randomly select 347 human proteins from UniHI as a reference set and calculate their median degrees. At each degree *k* in the range of 1-51, we calculate the percentage of proteins with degrees >= *k*, and plot the degree distributions of these three groups of proteins in Fig. 2a. We discover that at each degree *k* there are higher fractions of bioassay targets with degree >= *k* than is the case for drug targets and random proteins. Although it is difficult to speculate how a bias toward high interaction targets might have emerged, this significant difference in median degrees of bioassay targets and known drug targets (*P* < 10^–5^, Wilcoxon rank-sum test) has positive implications in that high degree proteins are more likely to participate in multiple pathways, and their modulation is thus likely to yield biochemical implications of scientific interest. The high degree also identifies targets as being somewhat distinct relative to the current body of drug targets and thus may theoretically afford novel avenues for eventual therapeutics development.

To gain better understanding of the biological functions and the potential applications of these bioassays, we need to map the original bioassay-compound bipartite network into real biological networks, such as drug-target network [2], human disease-gene network [15], and human protein interactome [9]. As subsets of real biological networks, these mapped networks can provide important clues for identifying potential drug targets with promising therapeutics to currently critical diseases. We first investigate how bioassay targets distribute around the essential genes. Picking an essential gene in the UniHI network, we calculate the shortest distances between it and other proteins. At each distance, we compute the fraction of bioassay targets in all proteins with the same shortest distance to the picked essential gene. We perform this shortest distance search on all essential genes, and get the mean fraction of bioassay targets over them at each distance. In addition, to test the significance of the results, we randomly pick a protein and repeat the same experiment for 10,000 times and obtain the mean fraction of bioassay targets around the random protein at each shortest distance.

The results are shown in Fig. 2b, which shows that the fractions of bioassay targets around essential genes are significantly higher than the randomized expectation at distance 1 and 2. At distance 1 from an essential gene, on average 5.28% proteins are bioassay targets, compare with the random expectation of 3.94% (*P* < 10^–101^). At distance 2, the percentage of bioassay targets is 3.63% and 3.17%(*P* < 10^–44^), respectively. These results confirm that bioassay targets are more clustering around essential genes than regular proteins, which is a positive sign for bioassay targets being potential drug targets according to Yildrim *et al.*[*2*]. In addition, we also perform the same experiments to investigate the clustering of bioassay targets around drug targets (Fig. 2c) and disease genes (Fig. 2d). Significant differences are observed between the normal test and random control test (At distance 1 from a bioassay target, *P* < 10^–24^ for drug targets and *P* < 10^–12^ for disease genes. At distance 2, *P* < 10^–19^ for drug targets and *P* < 10^–27^ for disease genes), showing that bioassay targets are also statistically closer to existing drug targets and disease genes than regular proteins in UniHI, especially for drug targets.

### Network models for BioAssay targets

The qualitative analysis above has answered “yes” to this question: “Are the PubChem bioassays going on the right track for identifying promising potential drug targets?” To gain more understanding on the potential of bioassay targets being new drug targets quantitatively, we develop a model to quantify the clustering of essential genes and drug targets surrounding them. First, by computing the fraction *f_i_* of essential genes at each distance *d_i_*, we obtain the distribution of bioassay targets around essential genes, and then define the characteristic distance  from an essential genes as follows: , where *n* is the diameter of the network. The characteristic distance  of an essential bioassay target will be less than 1.0, and that of non-essential bioassay targets will be close to or greater than 1.0. The mechanism underlying this formula is from the Coulomb law in electrostatics. We can view each “essential gene” as a unit charge that generated an electric field with field strength , and all these electric fields accumulate at the position of the picked bioassay target. We rank bioassay targets based on their  values, and the ranking quantifies the distance between a bioassay target and the essential genes surrounding it.

The characteristic distance  between bioassay targets and drug targets can be calculated with the same formula. We then rank the characteristic distance  of the remaining bioassay targets in the ascending order, and discretize  with bin size of 0.5 (e.g., [1.0,1.5), [1.5,2.0), …). Within each bin, bioassay targets are ranked by the descending order of the  values since we expect to find diverse new drug targets with longer distance from existing drug targets. Finally, bioassay targets with small degrees in the UniHI network are excluded. According to this paradigm, the top 10 potential drug targets with ,  and at least 17 interacting proteins are listed in Table 1. Literature survey confirmed that 7 of the top 10 bioassay targets are promising drug targets under investigation. The results demonstrate the correlation between the potential of bioassay targets as new drug targets and how close they are to essential genes and existing drug targets in the human PPI network.

**Table 1:** Summary of the calculation results for the top 10 predictions of bioassay targets and the literature that confirm them as potential drug targets.

Gene Symbol			Degree	Literature Evidence
SULT1E1	1.4744	5.1837	17	Confirmed [19]
WEE1	1.3499	3.1641	47	Confirmed [20]
RGS7	1.3440	3.0462	20	Confirmed [21,22]
SMN2	1.6241	5.8903	28	Confirmed [23]
RNGTT	1.7738	4.2353	100	Not yet
STK16	1.6785	3.9232	34	Not yet
PAK7	1.7091	3.8467	30	Confirmed [24]
NEK2	1.7952	3.5328	47	Confirmed [25]
YWHAG	1.8603	3.3451	339	Not yet
MAPK10	1.6685	3.0371	27	Confirmed [26]

drug targets, and (d) disease genes at each shortest distance in the human PPI network, compared with the corresponding randomized expectation.

## Conclusions

In this work we integrate PubChem bioassay data and other biological databases such as DrugBank and UniHI, and systematically analyze them to address these questions: 1) Are the present bioassays going on the right track for identifying new drug targets? 2) What are the relationships between bioassay targets and existing drug targets in the context of human protein interactome? 3) How to quantify the potential of bioassay targets being new drug targets? In addition to the basic science objective of producing chemical probes for studying the functions of genes, cells, and biochemical pathways at a molecular level, an original mandate of the MLI program has been to provide an informational basis to support drug discovery in service of important biomedical objectives [7]. Although not directly aligned with current MLI mandates, these questions may prove useful in gauging MLI progress as a cradle for fostering chemical biology research breakthroughs and future therapeutics discovery.

In this work, we first construct a bioassay network, and use network topology analysis to demonstrate that this is a scale-free network but is not hierarchical, which is different from typical biological networks that are usually both scale-free and hierarchical. Some cell-based bioassays share a large portion of active compounds with target-based bioassays, which is helpful to determine the interacting proteins in the cells. We map bioassay targets into the human PPI network called UniHI and find they are significantly clustering around drug targets and essential genes than randomized expectation. Hence current bioassay screenings were on the right track for identifying potential drug targets. We observe that the median degree of bioassay targets is significantly higher than not only the UniHI network median degree, but also the median degree of approved drug targets. More importantly, our network analysis also reveal that bioassay targets are much more likely to have direct physical PPIs with established drug targets than is the case for randomly selected genes within the UniHI network. This critical finding should trigger the attention of the community for reconsidering the selection of bioassay targets in a more rational matter. Finally, we propose a model to quantitatively prioritize the potential of bioassay targets as new drug targets, and conduct literature survey for confirming our predictions with reasonable accuracy. These observations could shed bright insights to future therapeutic discovery.

In conclusion, this paper represents an attempt to objectively assess the MLI progress to date as a tool for the chemical biology and pharmaceutical communities, by probing the relative novelty of target selection, the likelihood that these targets will prove interesting from a chemical biology or a potential therapeutic perspective. Significant distributional differences between bioassay targets and known drug targets speaks well of a basic science program introducing new insight into the field of chemical biology.

### Methods

#### Data sources

We obtain the chemical structures of all approved drug compounds and the gene symbols of their target proteins from the DrugBank database [12,13]. As of January 22, 2009, there are 1,493 FDA-approved drugs, more than 800 human proteins, and the drug-drug target associations. Data of human disease genes are downloaded from the OMIM database [14]. The resulting bipartite human disease network consisting of 1,284 distinct diseases, 1,777 disease genes, and 2,929 disease-gene associations. In addition, human PPI data are obtained from UniHI, a unified human PPI network containing over 250,000 human PPIs collected from 14 major PPI sources, including high-quality systematic interactome mapping and literature curation.

#### Mouse phenotype and human essential genes

A human gene is defined as “essential” if a knockout of its mouse ortholog confers lethality. To find human essential genes, we first extract mouse essential genes from the Mouse Genome Informatics Database [18], and through the human-mouse ortholog associations we obtain human essential genes, which correspond to 2,564 entries in UniHI. We obtain the official gene symbol of each bioassay target from its GI number, and find 228 entries among the total 21,051 human proteins in UniHI.

#### Network and topological analysis

A network is an undirected graph consisting of some nodes and edges connecting nodes. A node can represent any object, and an edge connects two nodes and usually carries some physical meaning such as interaction, similarity, and etc. The “degree” of a node is the number of edges connecting it to other nodes. Given a node degree *i*, we count the number of nodes with degree *i* in the network (*i* = 1, 2, …), and then normalize these counts to obtain the node degree distribution. The clustering coefficient *C_i_* of a network is defined as *C_i_* = 2*n/*[*k_i_* ∗ (*k_i_* – 1)], where *n* denotes the number of direct neighbors of a given node *i*, and *k_i_* is the number of links among the *n* neighbors of node *i*. If the clustering coefficient of a node equals 1, the node is at the center of a fully connected cluster called a clique. If the clustering coefficient is close to 0, the node is in a loosely connected region. We calculate average clustering coefficient over nodes with the same degree, and then obtain the distribution of average clustering coefficient over node degrees. The average of *C_i_* over all nodes of a network assesses network modularity. In this paper, the layouts of the projected bioassay network are obtained using free software called Cytoscape 2.6.2, and its free plug-in named Network Analyzer is used to compute all the network topological parameters and distributions.

#### Statistical tests

Wilcoxon rank-sum test is used to test if the medians of two sample vectors are equal, and returns the probability **P** of the positive answer at a given significance level (it is 0.05 in all tests performed in this paper). We call the “ranksum” function in the MATLAB software to conduct all Wilcoxon rank-sum tests.

## Methods

### Data sources

We obtain the chemical structures of all approved drug compounds and the gene symbols of their target proteins from the DrugBank database [12,13]. As of January 22, 2009, there are 1,493 FDA-approved drugs, more than 800 human proteins, and the drug-drug target associations. Data of human disease genes are downloaded from the OMIM database [14]. The resulting bipartite human disease network consisting of 1,284 distinct diseases, 1,777 disease genes, and 2,929 disease-gene associations. In addition, human PPI data are obtained from UniHI, a unified human PPI network containing over 250,000 human PPIs collected from 14 major PPI sources, including high-quality systematic interactome mapping and literature curation.

### Mouse phenotype and human essential genes

A human gene is defined as “essential” if a knockout of its mouse ortholog confers lethality. To find human essential genes, we first extract mouse essential genes from the Mouse Genome Informatics Database [18], and through the human-mouse ortholog associations we obtain human essential genes, which correspond to 2,564 entries in UniHI. We obtain the official gene symbol of each bioassay target from its GI number, and find 228 entries among the total 21,051 human proteins in UniHI.

### Network and topological analysis

A network is an undirected graph consisting of some nodes and edges connecting nodes. A node can represent any object, and an edge connects two nodes and usually carries some physical meaning such as interaction, similarity, and etc. The “degree” of a node is the number of edges connecting it to other nodes. Given a node degree *i*, we count the number of nodes with degree *i* in the network (*i* = 1, 2, …), and then normalize these counts to obtain the node degree distribution. The clustering coefficient *C_i_* of a network is defined as *C_i_* = 2*n/*[*k_i_* ∗ (*k_i_* – 1)], where *n* denotes the number of direct neighbors of a given node *i*, and *k_i_* is the number of links among the *n* neighbors of node *i*. If the clustering coefficient of a node equals 1, the node is at the center of a fully connected cluster called a clique. If the clustering coefficient is close to 0, the node is in a loosely connected region. We calculate average clustering coefficient over nodes with the same degree, and then obtain the distribution of average clustering coefficient over node degrees. The average of *C_i_* over all nodes of a network assesses network modularity. In this paper, the layouts of the projected bioassay network are obtained using free software called Cytoscape 2.6.2, and its free plug-in named Network Analyzer is used to compute all the network topological parameters and distributions.

### Statistical tests

Wilcoxon rank-sum test is used to test if the medians of two sample vectors are equal, and returns the probability **P** of the positive answer at a given significance level (it is 0.05 in all tests performed in this paper). We call the “ranksum” function in the MATLAB software to conduct all Wilcoxon rank-sum tests.

## Competing interests

The authors declare that there are no competing interests.

## Authors' contributions

The original basic idea was conceived by JH. JZ and GHL designed and implemented all analyses, tests, and experiments.
